# Therapeutic potential of a gamma-secretase inhibitor for hearing restoration in a guinea pig model with noise-induced hearing loss

**DOI:** 10.1186/1471-2202-15-66

**Published:** 2014-05-22

**Authors:** Yosuke Tona, Kiyomi Hamaguchi, Masaaki Ishikawa, Takushi Miyoshi, Norio Yamamoto, Kohei Yamahara, Juichi Ito, Takayuki Nakagawa

**Affiliations:** 1 Department of Otolaryngology, Head and Neck Surgery, Graduate School of Medicine, Kyoto University, Kawaharacho 54, Shogoin, Sakyoku, Kyoto 606-8507, Japan

**Keywords:** Cochlea, Hair cell, Notch signaling, Regeneration

## Abstract

**Background:**

Notch signaling plays a crucial role in the fate determination of cochlear progenitor cells, hair cells, and supporting cells in the developing cochlea. Recent studies have demonstrated the temporal activation of Notch signaling in damaged mature cochleae, and have demonstrated the induction of new hair cells by pharmacologically inhibiting Notch signaling. The present study aimed to illustrate the feasibility of pharmacologically inhibiting Notch signaling by using a gamma-secretase inhibitor for treating sensorineural hearing loss.

**Results:**

The effect of the sustained local delivery of MDL28170, a gamma-secretase inhibitor, on hearing and hair cell induction was tested in a guinea pig model with noise-induced hearing loss. MDL28170 was directly delivered into the cochlear fluids via a micro-osmotic pump. Drug application was initiated 7 days after noise exposure. Measurements of auditory brainstem responses revealed better hearing in the MDL28170-treated animals than in the vehicle controls. Histological analysis demonstrated a higher number of outer hair cells in the MDL28170-treated cochleae than the vehicle-treated cochleae.

**Conclusion:**

These findings strongly suggest that local sustained delivery of a gamma-secretase inhibitor into the cochlea could be a novel strategy for treating acute hearing loss that is refractory to conventional treatment.

## Background

Sensorineural hearing loss (SNHL) is a common disability. However, in general, hearing never returns once it is lost. There are no therapeutic options for SNHL, except hearing aids and cochlear implantation. The mammalian cochlea has virtually no regenerative capacity, which is a main reason for the difficulty in treating SNHL. Inducing regeneration of auditory hair cells, which convert sound stimuli into neural signals, has been a particularly critical issue in developing novel therapeutic strategies for SNHL.

The Notch signaling pathway is involved in the cell fate determination of various cell lineages [1-7]. This signaling pathway exerts its effect when the interaction of the Notch receptor and its ligand induces proteolytic cleavage of the Notch receptor through gamma-secretase activity [8,9]. A gamma-secretase inhibitor achieves pharmacological inhibition of Notch signaling by taking advantage of this mechanism [10,11]. Previous studies regarding the developmental biology of the inner ear have demonstrated the important role of Notch signaling in cell fate specification into hair cells or supporting cells from cochlear prosensory cells [12-15]. In the cochlear epithelium, the activation of Notch signaling resulted in the upregulation of Hes1 and Hes5 leading to differentiation of progenitor cells into supporting cells, while inactivation of Notch signaling induced the expression of Atho1, which is one of the key molecules for hair cell differentiation. Previous studies have demonstrated that the overexpression of Atho1 in cochlear epithelia induces hair cells from supporting cells [16-19]. When Notch signaling is inhibited genetically [12,15] or pharmacologically [11] during the cochlear developmental stage, an increased number of hair cells and a decreased number of supporting cells are observed. Based on these observations, several studies attempted the induction of new hair cells in the postnatal stage when the cell fate specification has already ceased [10,20]. Genetic and pharmacological inhibition of Notch signaling causes ectopic hair cell induction in the neonatal mouse cochlea [10]. Moreover, a recent report has shown that the pharmacological inhibition of Notch signaling induces the generation of new hair cells, even in mature mouse cochleae, by the transdifferentiation of supporting cells [20].

Based on findings in explant cultures of neonatal mouse cochleae [10], we previously examined the potential of MDL28170 (MDL), a gamma-secretase inhibitor, for the induction of new hair cells in adult guinea pig cochleae in vivo [21]. In that study, an ototoxic treatment that caused the total loss of the outer hair cells was used to aid the identification of newly generated hair cells. As a result, the induction of new hair cells was identified, although their number was limited. The severe degeneration of supporting cells (a source of newly generated hair cells) may be the reason for the poor induction of new hair cells. On the other hand, single application of the gamma-secretase inhibitor LY411575 into the middle ear was recently tested in a mouse model with noise-induced hearing loss; the treatment resulted in the induction of new hair cells and in hearing recovery [20]. However, a high activity of LY411575 for the inhibition of Notch signaling involves a risk of adverse events, in particular promotion of malignancies [22]. Therefore, as an alternative for LY411575, a gamma-secretase inhibitor with moderate, but sufficient, activity for the inhibition of Notch signaling is needed for clinical application.

The present study aimed to test the potential of sustained local delivery of MDL into cochlear fluids for the induction of new hair cells and the recovery of hearing in a guinea pig model with a previously established noise-induced hearing loss [23]. In this model, apoptosis of the outer hair cells in selective regions of the cochlea occurred within 5 days after the noise exposure; the supporting cells were preserved [23]. To examine the potential of inducing new hair cells after hair cell loss, local application of MDL was initiated 7 days after the noise exposure [23]. For the sustained delivery of MDL, a micro-osmotic pump was used, as described in previous studies [21,23]. In the present study, the MDL-treated animals exhibited better hearing than the vehicle control animals and had higher numbers of outer hair cells in the cochleae than the vehicle control animals. These findings strongly suggest the potential of gamma-secretase inhibitors as therapeutics for the treatment of SNHL.

## Methods

### Animals

Hartley strain guinea pigs weighing 350–400 g were purchased from Japan SLC, Inc. (Hamamatsu, Japan). The Animal Research Committee of the Graduate School of Medicine at Kyoto University approved all experimental protocols. Animal care was supervised by the Institute of Laboratory Animals of the Graduate School of Medicine at Kyoto University. All experimental procedures were performed in accordance with the National Institutes of Health Guide for the Care and Use of Laboratory Animals. All efforts were made to limit the number of animals used and their suffering.

### Noise exposure

Guinea pigs were exposed to a continuous pure tone at 6 kHz with a 130-dB SPL for 15 min under general anesthesia [23]. Each animal was immobilized in a noninvasive head holder. A speaker was positioned 10 cm in front of the animal’s head. Sound levels were monitored using a 1/2 inch condenser microphone (Sony, Tokyo, Japan) and a fast Fourier transform analyzer (Sony).

To clarify the cochlear damage before the drug application, cochlear specimens (n = 4) were collected on day 7 and were processed histologically.

To determine noise-induced damage, ABR recording was performed 7 days after noise exposure. Animals showing 40 dB and larger ABR threshold shifts in both ears and smaller than 10 dB differences in threshold shifts between left and right ears were provided following experiments. To determine symmetrical lesions in both ears of animals that matched this condition, we compared hair cell numbers in left ears with those of right ears (n = 4).

### Local drug application

On day 7 after the noise exposure, the gamma-secretase inhibitor MDL (Sigma-Aldrich, St. Louis, MI) was locally applied to the perilymph. Under general anesthesia, a cochleostomy was performed 1 mm from the edge of the round window on the basal turn of the cochlea. A Tefron tube with an inner diameter of 180-μm (UT-03, Unique Medical Co., Ltd., Tokyo, Japan) was connected to a micro-osmotic pump (Alzet, Cupertino, CA) and inserted into the scala tympani of the basal turn of the left cochlea of each guinea pig. The mini pump pumped MDL at a rate of 0.25 μL/h for 14 days. The tip of a Tefrone tube positioned at distance of 3 mm from the round window membrane, which accords with the 85% region from the cochlea apex [24], in order to deliver MDL to a damaged region, 70–80% region from the apex securely.

The MDL was dissolved in dimethyl sulfoxide (DMSO) and diluted with a phosphate buffered saline (PBS) to give a final concentration of 1 mM containing 0.3% DMSO. This concentration was also used in our previous work [21]. For 14 days, the MDL solution was continuously injected through the micro-osmotic pump into the left cochlea of the guinea pigs (n = 7). The control animals (n = 5) received PBS (instead of MDL solution) into the left cochlea; the PBS contained 0.3% DMSO. In addition, to examine the effects of MDL treatment on the normal cochlear epithelia of mature guinea pigs, normal guinea pigs (n = 3) received local a MDL application through the micro-osmotic pump.

To examine cell proliferation in the cochlear sensory epithelium by MDL treatment, the assay for labeling with 5-ethynyl-2′-deoxyuridine (EdU) was performed. EdU (Click-iT® EdU Imaging Kits, Molecular Probe, Eugene, OR) was added to the MDL solution (n = 4) or the vehicle solution (n = 4). The final concentration of EdU was 10 μM.

### Functional assessments

Auditory function was assessed by recording auditory brainstem responses (ABRs) and distortion-product otoacoustic emissions (DPOAEs). The baseline ABR thresholds were measured within 7 days before the noise exposure. The ABR thresholds were measured at frequencies of 8, 10 and 12 kHz, which correspond to the regions damaged by noise trauma used in this study [24]. The animals were anesthetized with an intramuscular injection of midazolam (10 mg/kg) and an intramuscular injection of xylazine (10 mg/kg). They were kept warm with a heating pad. The generation of acoustic stimuli and the subsequent recording of evoked potentials were performed using the Powerlab/4sp data acquisition system (ADInstruments, Colorado Springs, CO, USA), as described in our previous study [25]. Acoustic stimuli, consisting of tone-burst stimuli (0.1 ms cos 2 rise/fall and 1-ms plateau), were delivered monaurally through a speaker (ES1spc; Bioresearch Center, Nagoya, Japan) connected to a funnel fitted into the external auditory meatus. The thresholds were determined from a set of responses at varying intensities with 5-dB sound pressure level (SPL) intervals. The electrical signals were averaged over 1024 repetitions. The thresholds at each frequency were verified at least twice. ABR recordings were performed 14 days after drug application. In comparison ABR threshold shifts in MDL-treated cochleae with those in vehicle-treated cochleae, values that ABR threshold shifts in drug-treated cochleae (left) were subtracted those of contralateral cochleae (right) to reduce influences due to individual differences.

DPOAE recordings were made with an acoustic probe (ER-10C; Etymotic Research, Elk Grove Village, IL) using the DP2000 DPOAE measurement system version 3.0 (Starkey Laboratory, Eden Prairie, MN) before sacrifice. Two primary tones with an f2/f1 ratio of 1.2 were presented at intensity levels of 65 dB SPL (L1) and 55 dB SPL (L2). The f2 was varied in one-ninth-octave steps from 8 to 12 kHz. A peak at 2f1–f2 in the spectrum was recognized as a DPOAE. The DP/noise floor (NF) levels were calculated.

### Histological analysis

On day 14 after drug application, the Tefron tube was gently removed from the cochlea under general anesthesia. Finally, MDL-treated (n = 3), vehicle-treated (n = 3), MDL + EdU-treated (n = 3) and vehicle + EdU treated cochleae (n = 3) were obtained. After removing the stapes from the oval window, followed by opening the cochlear apex, 4% of paraformaldehyde in PBS was perfused into the cochlea. The excised cochleae were then immersed in the same fixative at 4°C for 12 h. After decalcification with 0.1 M ethylenediaminetetraacetic acid for 14 days at 4°C, the cochleae were subjected to histological analysis of whole mounts. The cochlear specimens were permeabilized in 0.2% Triton X in PBS for 30 min at room temperature. To determine the location of the hair cells, immunohistochemistry was performed for myosin VIIa (anti-myosin VIIa rabbit polyclonal antibody; 1:500; Proteus Bioscience Inc., Romana, CA) and F-actin labeling by fluorescein-phalloidin (1:400; Molecular Probes). At the end of the staining procedures, nuclear staining was performed with 1 μg/mL of 4′,6-diamidino-2-phenylindole (DAPI) (Molecular Probes) in PBS. For an EdU assay, a Click-iT® EdU Imaging Kit (Molecular Probes) was used. Staining was performed according to the supplier’s direction followed by immunostaining for myosin VIIa and DAPI staining. Specimens were viewed with a Leica TCS SP2 confocal microscope (Leica Microsystems Inc., Wetzlar, Germany).

### Cell counts

Quantitative analyses for hair cell numbers were performed in the 70–80% region from the apex of the cochlea. The cells that were positive for myosin VIIa with phalloidin-labeled stereocilia and DAPI-positive nuclei were defined as surviving hair cells. The number of inner hair cells and outer hair cells was counted respectively. Considering individual differences between animals, we subtracted hair cell numbers of right cochleae from those of left cochleae that were locally applied drugs.

### Statistical analyses

An unpaired *t*-test (one-tailed) was used to compare differences between the groups, and a paired *t*-test (one-tailed) was used for comparisons between left and right cochleae. The data are presented in the text and figures as the mean ± the standard error of the mean (SEM). A *p* value less than 0.05 was considered significant.

## Results

### Noise-induced damage

**Figure 1:**
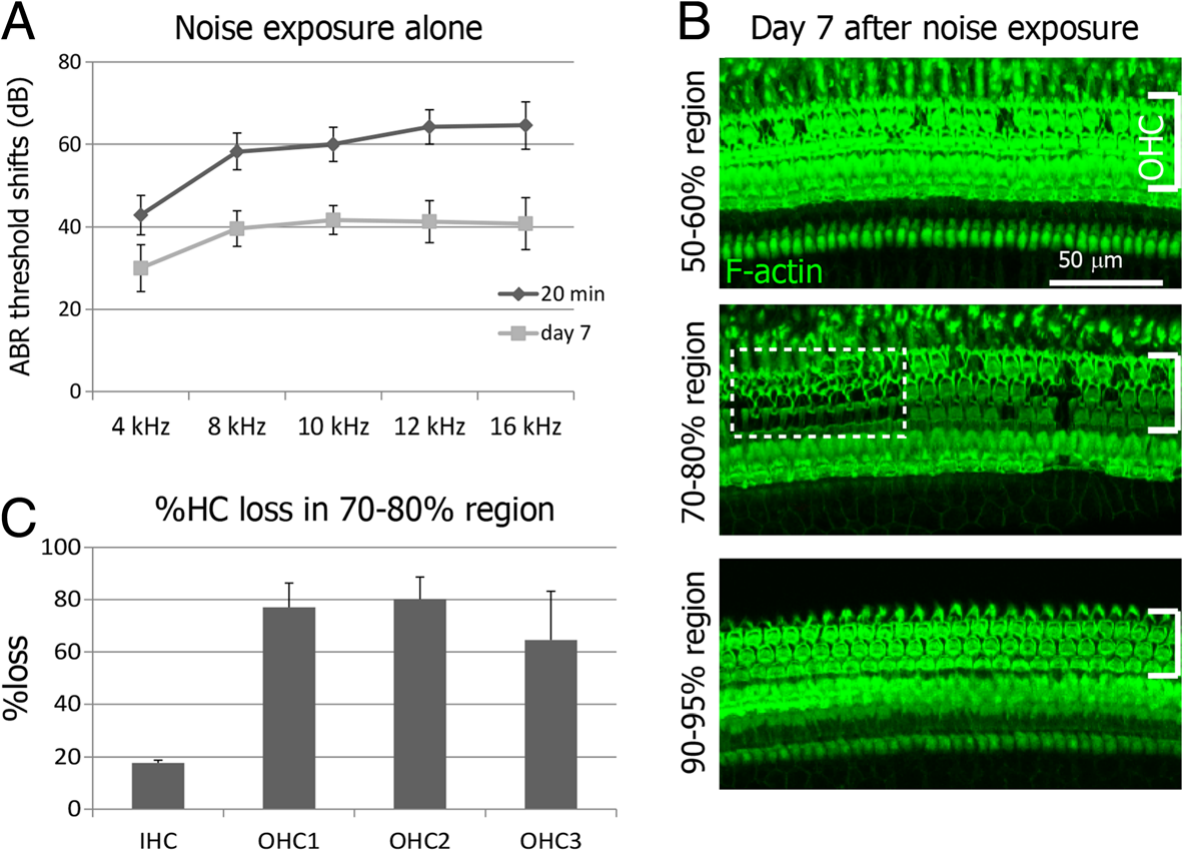
**Functional and histological damage in guinea pig cochleae by noise exposure. A**: ABR threshold shifts 20 min and 7 days after noise exposure in guinea pigs (n = 4). **B**: F-actin labeling with phalloidin in cochlear epithelia in the 50–60%, 70–80% and 90–95% regions from the cochlear apex. Brackets indicate the location of the outer hair cells. A square of dotted lines shows a patch of severe hair cell loss. **C**: The percent loss of hair cells in the inner hair cells (IHC), or in the first row (OHC1), second row (OHC2), or third row (OHC3) of the outer hair cells in the 70–80% region of cochlear epithelia from the apex.

**Figure 2:**
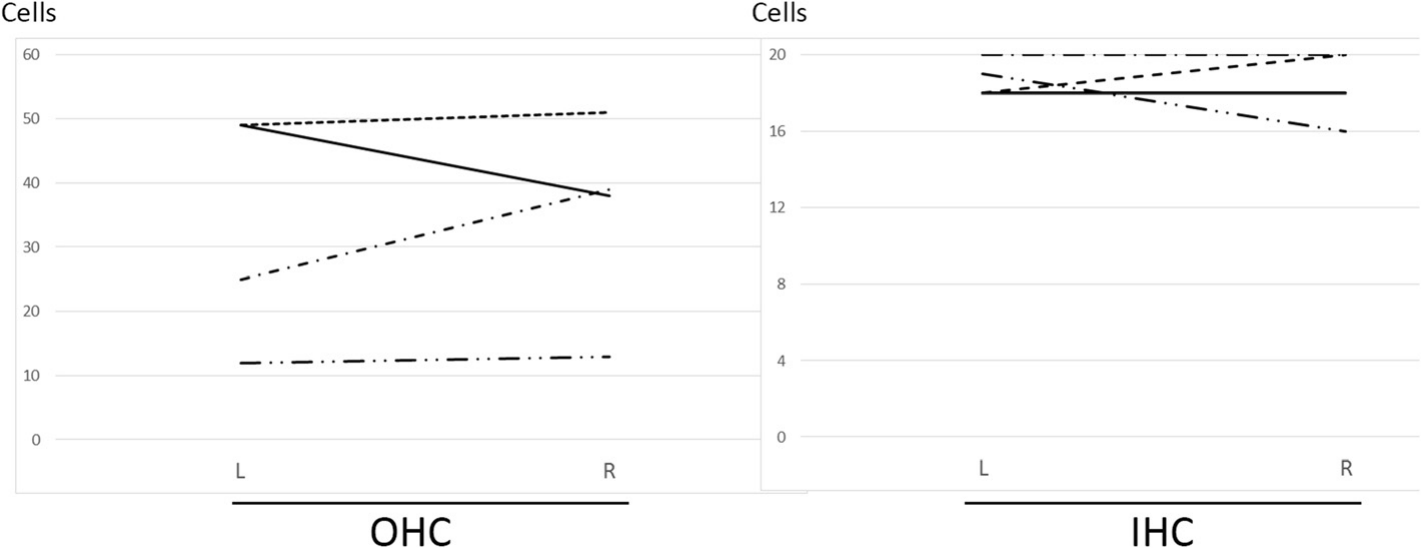
**Comparison of hair cell numbers in left and right ears of animals matched ABR criteria for noise-induced damage.** Remaining numbers of outer (OHC) and inner hair cells (IHC) were nearly symmetrical in both ears (n = 4). There were no significant differences in numbers of OHC or IHC between left (L) and right (R) ears with paired t-test.

Noise exposure used in this study was expected to induce selective loss of outer hair cells in the 70–80% region from the apex of the guinea pig cochlea, as previously described [23]. To test the reproducibility of hair cell loss in the present study, cochlear specimens obtained on day 7 after the noise exposure were examined histologically (n = 4). In the 50–60% region from the apex of cochleae, only a scattered loss of outer hair cells was identified, but the inner hair cells were well preserved (Figure 1). In the 90–95% region from the apex of the cochlea, the inner and outer hair cells were well preserved (Figure 1). On the other hand, as we expected, a massive loss of outer hair cells was detected in the 70–80% region from the apex of the cochleae (Figure 1). There was one patch or two patches of severe outer hair cell loss surrounded by areas of scattered outer hair cell loss (Figure 1). This pattern of hair cell loss is almost identical to previous findings by using the same noise exposure [23].On day 7 after noise exposure, hair cell numbers of the left cochleae were compared with those of the right cochleae in four animals matched the criteria for hearing loss (Figure 2). The lesions were almost symmetrical in both sides. There were no significant differences in numbers of inner or outer hair cells between left and right cochleae.

### ABR threshold shifts

**Figure 3:**
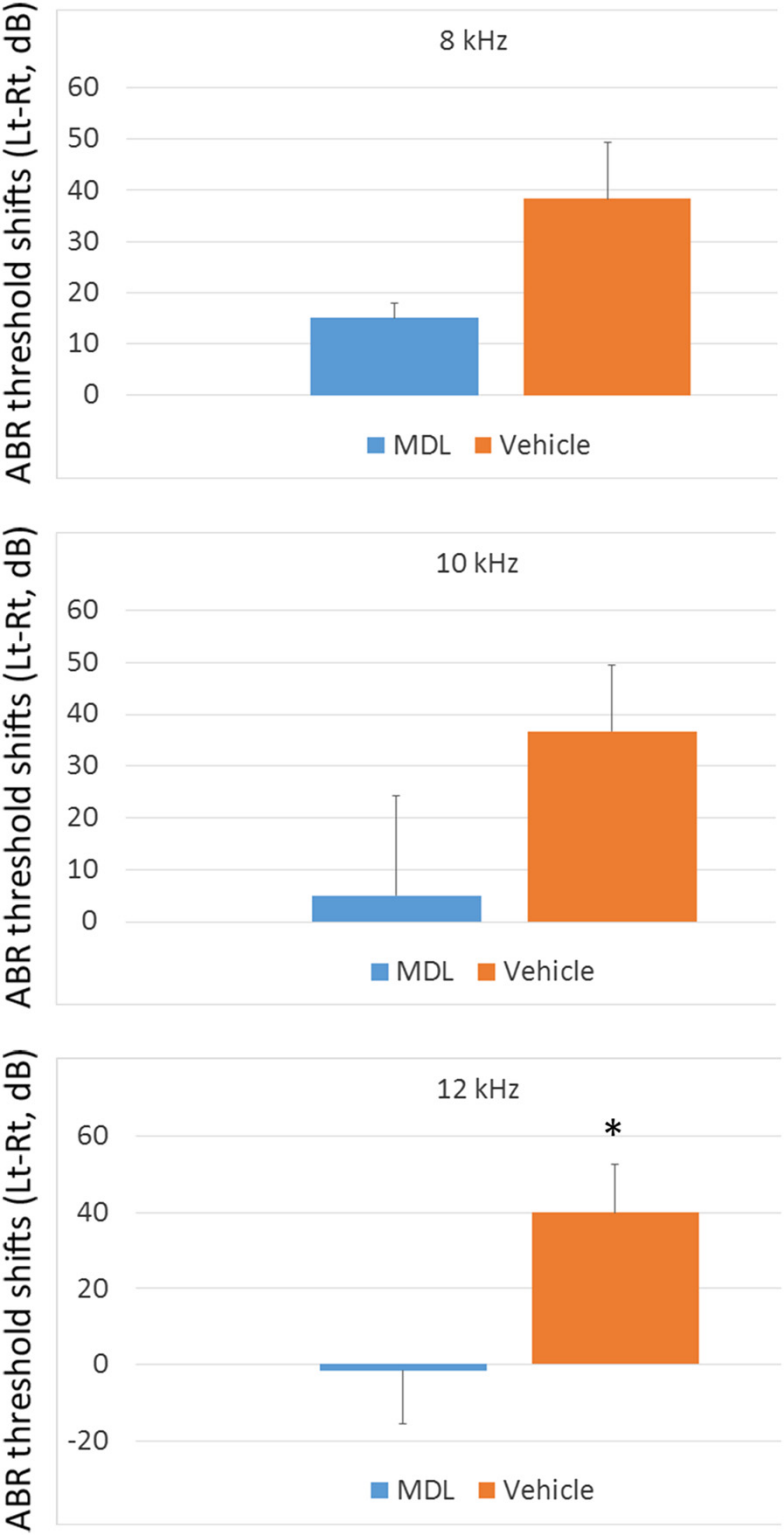
**ABR threshold shifts compared with contralateral ears in MDL- and vehicle-treated animals.** ABR threshold shifts of MDL- or vehicle-treated ears subtracted those of contralateral ears are represented. Bars represent SEM. Differences in ABR threshold shifts between MDL- (n = 3) and vehicle-treated (n = 3) ears are statistically significant at p < 0.05 with unpaired t-test at 12 kHz (*).

We measured the ABR threshold shifts at frequencies of 8 kHz, 10 kHz and 12 kHz, because these frequencies correspond to the 70–80% region from the apex of the guinea pig cochlea [24]. ABR threshold shifts in drug-treated cochleae (left) were subtracted those of contralateral cochleae (right) to reduce influences due to individual differences, which were defined as corrected ABR threshold shifts. We compared corrected ABR threshold shifts between MDL- and vehicle treated cochleae (Figure 3). At 12 kHz, MDL-treated cochleae exhibited significantly smaller ABR threshold shifts than vehicle-treated cochleae (unpaired t-test, p = 0.04).

### DPOAEs

**Figure 4:**
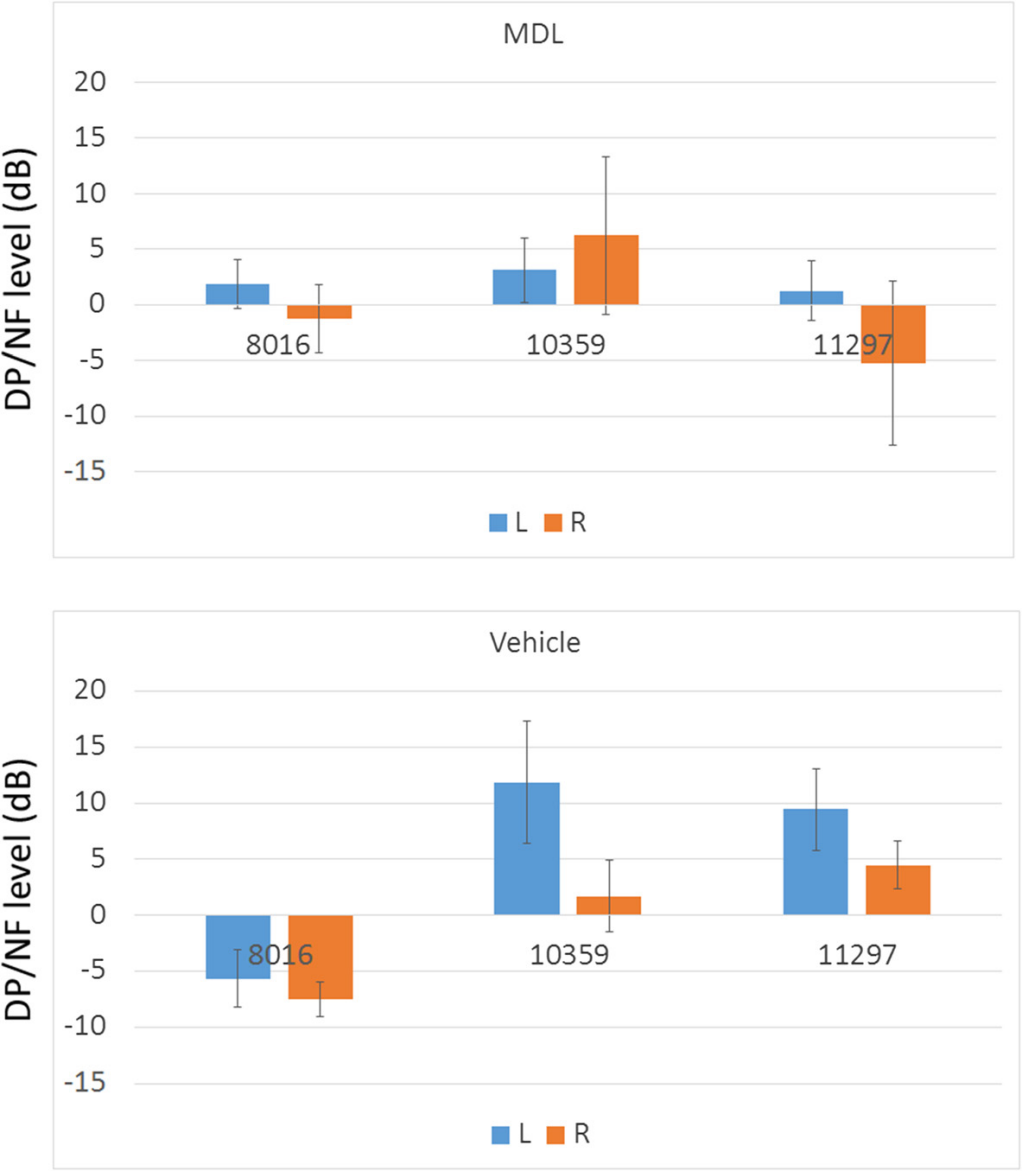
**DPOAE in MDL- and vehicle-treated animals.** DP/NF levels of both ears in MDL- and vehicle-treated animals are shown. X-axis shows F2 frequency (Hz). No significant differences in DP-NF levels between left (L) and right (R) ears of MDL- or vehicle-treated animals, and between MDL- and vehicle-treated ears.

DPOAE measurements were performed 14 days after drug application. DP/NF levels in MDL- and vehicle-treated animals are shown in Figure 4. No significant differences in DP/NF levels between left and right ears in either group, or between MDL- and vehicle-treated cochleae.

### Histology of cochlear epithelia

**Figure 5:**
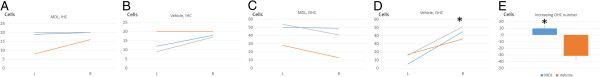
**Numbers of outer hair cells in MDL- and vehicle- treated animals.** Left ears were locally applied drugs. **A**: Numbers of inner hair cells (IHC) in left (L) and right (R) cochleae of MDL-treated animals. **B**: Numbers of IHC in left (L) and right (R) cochleae of vehicle-treated animals. **C**: Numbers of outer hair cells (OHC) in left (L) and right (R) cochleae of MDL-treated animals. **D**: Numbers of OHC in left (L) and right (R) cochleae of vehicle-treated animals. The difference in OHC numbers between left and right in vehicle-treated animals was statistically significant at p < 0.05 with paired t-test (*). **E**: Increasing numbers of OHC in MDL- and vehicle-treated cochleae. The difference in increasing OHC numbers between MDL- and vehicle-treated cochleae was statistically significant at p < 0.05 with unpaired t-test (*).

**Figure 6:**
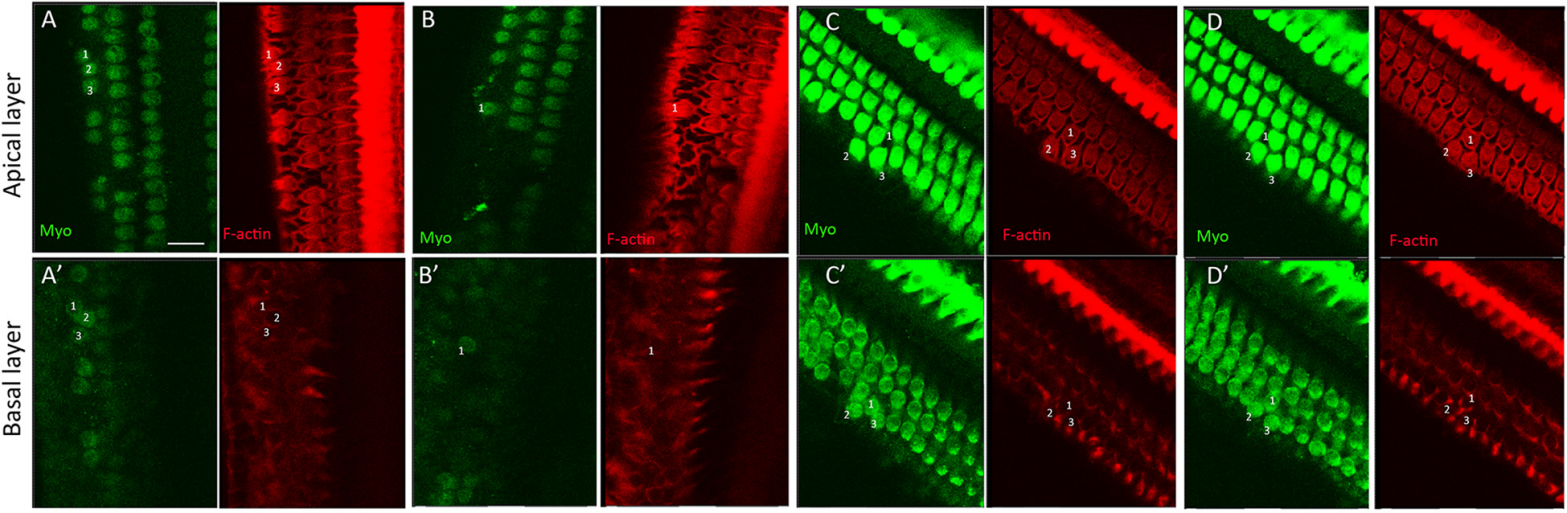
**Junctions between outer hair cells and Deiters cells in MDL-treated cochleae. A**-**D**: Focal plane at the apical region of outer hair cells. **A**’-**D**’: Focal plane at the basal portion of outer hair cells and the cups of Diters cells. In the areas adjacent severely damaged regions **(A, B)**, outer hair cells numbered 1 lacked junctions with the cups of Deiters cells **(A’, B’)**, while outer hair cells numbered 2 and 3 **(A’)** attached to the cups. In moderately damaged regions, excessive outer hair cells were frequently observed **(C, D)**. Such outer hair cells (numbered 2 and 3 in **C**, **D**) were connected with the cups of Deiters cells **(C’, D’)**. Scale bar in A represents 20 μm.

In both MDL- and vehicle-treated animals, inner hair cells were well preserved in both ears (Figure 5A, B). There were no significant differences in numbers of inner hair cells between left and right ears in MDL- or vehicle-treated animals, and between MDL- and vehicle-treated cochleae. In contrast to inner hair cells, degeneration of outer hair cells was found in all specimens. One of three animals treated with MDL exhibited extensive loss of outer hair cells (Figure 3B). In vehicle-treated cochleae, all specimens showed severe loss of outer hair cells, while in contralateral cochleae, outer hair cells were comparatively well preserved (Figure 3D). The difference in numbers of outer hair cells between left and right ears in vehicle-treated animals was significant (paired t-test, p = 0.02). We compared degeneration of outer hair cells in MDL-treated cochleae with that in vehicle-treated cochleae using subtraction differences in numbers of outer hair cells between drug-treated and contralateral cochleae (Figure 5E). Corrected numbers of outer hair cells in MDL-treated cochleae were significantly higher than those in vehicle-treated cochleae (unpaired t-test, p = 0.002).To clarify generation of new hair cells via transdifferentiation of supporting cells, we examined the presence of the cup of Deiters cell adjacent the base of outer hair cells. In a focal plane at the apical surface of the outer hair cell, F-actin labeling with phalloidin clearly showed the cuticular plates of outer hair cells (Figure 6A-D). Strong expression of myosin VIIa was also observed in this focal plane (Figure 6A-D). Thereafter, by putting the focal plane down to the level of the base of the outer hair cell, the cups of Deiters cells was demonstrated by F-actin labeling with pahlloidin, and the body of outer hair cells was labeled with myosin VIIa (Figure 6A’-D’). In the areas apical to severely damaged regions, excessive outer hair cells were observed in MDL-treated cochleae (Figure 6C-D). We confirmed the presence of the cups of Deiters cells beneath the base of outer hair cells (Figure 6C’, D’). On the other hand, in the areas adjacent severely damaged regions, the cups of Deiters cells were not identified beneath the base of outer hair cells in a few cases (Figure 6A’, B’). Consecutive observation of outer hair cells labeled with myosin VIIa along the z-axis indicated the existence of outer hair cells with long cell bodies (Figure 6B’). These findings suggested the occurrence of hair cell generation via transdifferentiation of supporting cells in the area adjacent severely damaged regions.

For evaluation of hair cell generation through proliferation of supporting cells, we employed the EdU assay. EdU was continuously applied to the cochlear fluid via a micro-osmotic pump for two weeks following noise exposure. Observation through a confocal microscope revealed the absence of EdU-positive cells within the cochlear sensory epithelia of MDL- or vehicle treated cochleae, indicating that no cell proliferation occurred in cochlear sensory epithelia after noise exposure and that MDL induced no cell proliferation on damaged cochlear sensory epithelia.

## Discussion

The gamma-secretase inhibitor has gained considerable attention because of its actions in inhibiting Notch signaling and amyloid beta-peptide formation. Because of the latter action, gamma-secretase inhibitors have been a top target for developing Alzheimer’s disease therapeutics [22]. On the other hand, the activity of gamma-secretase inhibitors for inhibiting Notch signaling has been an obstacle in the development of Alzheimer’s disease therapeutics because it is associated with in vivo toxicity [22]. In contrast, for the induction of hair cells from supporting cells, inhibiting Notch signaling is a crucial component of gamma-secretase inhibitor activity. With regard to clinical application, moderate, but sufficient, activity in inhibiting Notch signaling can be included in key issues for choosing compounds for local application to the cochlea.

Despite the short-term and weak activity of MDL for hair cell induction [10,16], local MDL application via a micro-osmotic pump resulted in better hearing recovery than did the application of the vehicle. A significantly higher number of outer hair cells were histologically observed in the MDL-treated cochleae than in the vehicle-treated cochleae. In the present study, MDL was applied to the damaged cochleae after hair cell loss had occurred. Morphological observation of MDL-treated cochleae demonstrated the presence of outer hair cells lacking the connection with the cups of Deiters cells. These findings suggest that induction of hair cells from supporting cells contributed to hair cell preservation in MDL-treated cochleae. However, direct evidence for trans-differentiation of supporting cells into hair cells, which was shown in previous studies [18,20], was not demonstrated in the present study. MDL is also known as a calpain inhibitor. Therefore, the protection of original outer hair cells may be involved in the mechanism for hair cell preservation with the MDL treatment [23]. Actually, the difference in numbers of outer hair cells between left and right cochleae in MDL-treated animals was not significant. In addition, greater loss of outer hair cells in vehicle-treated cochleae was found in comparison with that in contralateral cochleae. These findings suggest that direct drug application through a micro-osmotic mini pump may causes damage on outer hair cells and that MDL may rescue outer hair cells from damages associated with direct drug application to the cochlea. On the other hand, previous studies have demonstrated that cell proliferation did not contribute to an increase in hair cell numbers with a gamma-secretase inhibitor by a bromodeoxyuridine incorporation assay [11]. Present findings in an EdU assay demonstrated no occurrence of cell proliferation following MDL application. Therefore, we believe that mitosis in the cochlear epithelia may not be involved in the mechanism for preservation of hair cells in MDL-treated cochleae.

In the present study, local MDL application by using a micro-osmotic pump showed limited recovery of auditory function. DPOAE recording demonstrated no significant increase of DP/NF levels in MDL-treated cochleae. A possible explanation is that the function of newly generated or protected hair cells in the MDL-treated cochleae is inferior to that in the normal hair cells. In a previous study using LY411575, robust newly generated hair cells were observed in the LY411575-treated cochlea, while the functional gain of LY411575-treated cochlea was limited to 8 dB in ABR thresholds compared with controls [20]. Hair cell induction from supporting cells results in the loss of supporting cells, which could also affect auditory function. Present findings also demonstrated a problem due to direct drug application via a micro-osmotic mini pump, which affected the survival of outer hair cells. This may be resolved by the development of drug delivery systems using biodegradable materials. The functionality of newly generated or preserved hair cells and the influence of supporting cell loss because of hair cell induction should be investigated in the near future.

The present study results indicate that the use of topical drug application may compensate for the weak activity in the inhibition of Notch signaling. Therefore, compounds with weak activity in the inhibition of Notch signaling could be candidates for the treatment of hearing loss. The exploration of new therapeutics for Alzheimer’s disease has led to the discovery of numerous gamma-secretase inhibitors. The present findings encourage us to perform additional investigations in order to find the most appropriate one for clinical use. Together with the screening of gamma-secretase inhibitors for hearing recovery, the development of a drug delivery system that is specialized for a gamma-secretase inhibitor may be necessary to progress toward a clinical application.

## Conclusions

The present results demonstrated the preservation of hair cells and auditory function by local MDL application, indicating that even gamma-secretase inhibitors with a low activity for inhibiting Notch signaling may be candidates for the treatment of acute SNHL when appropriate drug delivery is achieved.

## Abbreviations

ABR: Auditory brainstem responses; DPOAE: Distortion-product otoacoustic emission; DAPI: 4′,6-diamidino-2-phenylindole; DMSO: Dimethyl sulfoxide; EdU: 5-ethynyl-2′-deoxyuridine; IGF-1: Insulin-like growth factor-1; MDL: MDL28170; PBS: Phosphate buffered saline; SNHL: Sensorineural hearing loss; SPL: Sound pressure level.

## Competing interests

The authors declare that they have no competing interests.

## Authors’ contributions

YT, KM, MI, TM and KY performed experiments. TN, NY and JI designed this study. YT, KM, MI, KH, TN and NY analyzed the data. TN and NY wrote the article. All authors read and approved the final manuscript.
